# Examining Patterns of Food Bank Use Over Twenty-Five Years in Vancouver, Canada

**DOI:** 10.1007/s11266-018-0039-2

**Published:** 2018-10-01

**Authors:** Jennifer L. Black, Darlene Seto

**Affiliations:** 1 Faculty of Land and Food Systems, Food, Nutrition and Health, University of British Columbia, 2205 East Mall, Vancouver, BC V6T 1Z4 Canada; 2 Greater Vancouver Food Bank, Vancouver, Canada

**Keywords:** Food banks, Emergency food system, Canada, Cluster analysis

## Abstract

Food banks have grown substantially in Canada since the 1980s but little is known about patterns or predictors of engagement including frequency or duration of service use. This study examined food bank program data from a large food bank organization in Vancouver, Canada, finding that between January 1992 and June 2017, at least 116,963 individuals made over 2 million food bank visits. The majority of members were engaged for a short time and came for relatively few visits, but 9% of members engaged in longer-term episodic or ongoing usage over several years, accounting for 65% of all visits. Results from cluster and regression analyses found that documented health and mobility challenges, larger household size, primary income source, and older age were predictors of higher frequency and duration of service usage. Findings add to growing critical examinations of the “emergency food system” highlighting the need for better understanding of the broader social policies influencing food bank use.

## Introduction

Similar to reported rising trends from the USA, the UK, and Australia (Booth and Whelan 2014; Riches and Tarasuk 2014; Caraher and Furey 2018a, b; Loopstra 2018), Canadian food banks have proliferated widely since the 1980s, and in 2016 were estimated to serve over 850,000 people on a monthly basis (Food Banks Canada 2016). In Canada, the term food bank is used to broadly describe organized, charitable food programs that provide a few days’ worth of supplemental food, usually at no cost, for individuals and families to take home and prepare (in contrast to meal or snack programs, where prepared or “ready-to-eat” food is provided). Similar charitable programs are also referred to elsewhere as food pantries, depots, and food shelves. Such programs have often been publicly framed as short-term benevolent responses aimed at helping individuals get back on their feet in times of acute financial challenges such as economic recessions or job loss (Tiehen 2002; Lambie-Mumford 2012; Cabili et al. 2013; Feeding America 2014).

Together with meal programs, food banks are commonly referred to as “emergency food providers” with little definition around the explicit meaning or anticipated duration of “emergency” use. Although food banks often frame their work around supporting clients’ immediate food needs, others are suspected to be filling chronic gaps left unmet by a public safety net increasingly buffeted by the forces of globalization, labour market transformations, and precarious employment (Riches 2002; Lightman et al. 2008; Caraher and Furey 2018a, b; Riches 2018). Some food banks explicitly aspire to provide services beyond collecting and distributing foods premised upon ongoing relationships or engagement with food bank users. For example, in addition to food, some food banks provide (or facilitate access to) health promotion and education services, life skills training, urban agricultural initiatives, among other programmatic activities (Riches 2002; McCullum et al. 2005; Martin et al. 2012; Wakefield et al. 2012; Loopstra et al. 2015; Roncarolo et al. 2016). Individual food banks, and the broader communities supporting these organizations may therefore maintain divergent philosophies and expectations about the characteristics and needs of clients served and the expected duration and frequency of their engagement with available services.

In Canada, food banks operate with limited public oversight and no representative governmental data are available characterizing patterns of population-level use. The most commonly cited sources of data about food bank usage (both in the USA and Canada) draw from cross-sectional reports estimating the number of total food banks or users in a given context, usually generated from a single or short span of time. In Canada, such data are collected by the non-profit association, Food Banks Canada, who conduct an annual assessment of the number of individuals receiving foods from affiliated food banks through the month of March. In 2016, over four thousand organizations participated in this study known as the “Hunger Count” (Food Banks Canada 2016). In the USA, a report called “Hunger in America” is generated by the national Feeding America organization. Between October 2012 and January 2013, a probability sample drawn from 32,677 agencies surveyed 60,122 clients (response rate = 61.9%) and weighted estimates suggested that 46.5 million people accessed a food bank each year in their network alone (Feeding America 2014). While both the national US and Canadian reports provide valuable snapshots about the scale of food bank use and characteristics and challenges of people who draw upon these services, they provide little insight regarding the frequency, intensity, or trajectories of service use over time or how usage patterns are impacted by the changing supply of or demand for services.

At the macro-systems level, food bank availability and proliferation is embedded within broader structural factors that shape inadequate or insecure access to food because of financial constraints (i.e. food insecurity), social welfare, and related social policies, but also corporate policies and structures that incentivise the redistribution of food through charitable giving. These topics are the focus of in-depth scholarly interest elsewhere, see for example: (Poppendieck 1998; Riches and Silvasti 2014; Fisher 2017; Riches 2018). Still, little available literature has examined if and how person-level characteristics and life circumstances shape more frequent or longer engagement. This study therefore explored measured individual characteristics commonly reported in cross-sectional studies of food bank users with plausible mechanisms for impacting long-term usage.

For example, evidence suggests that food bank users have commonly experienced protracted poverty, low incomes, unemployment, adverse life events and health challenges (Garthwaite et al. 2015; Loopstra and Lalor 2017; Prayogo et al. 2017; Holmes et al. 2018a, b). The majority of Canadians relying on social assistance report food insecurity (Tarasuk et al. 2016), and 45% and 18% of Canadian food bank users reported drawing on social assistance and disability-related income supports, respectively, in the 2016 Hunger Count survey (Food Banks Canada 2016). A recent mixed methods study of Canadian food bank users suggests that financial constraints related to low employment income and insufficient benefits from social assistance and disability benefits, as well as high housing costs and health challenges buttressed participants’ views of food banks as a long-term food augmentation strategy (Holmes et al. 2018a, b).

Household composition including living alone and in lone parent families can further increase susceptibility to food insecurity and likelihood of drawing on food banks (Loopstra and Lalor 2017; Prayogo et al. 2017; Holmes et al. 2018a, b). In Canada, nearly equal proportions of men and women draw on food banks (Food Banks Canada 2016), but little is known about how gender shapes perceived experiences or usage patterns. Life course stage and age could also shape service usage, with evidence of growing usage of homeless shelters among Canadians over age 50 between 2004 and 2014 (Government of Canada 2016a, b). Nationally, seniors over 65 years are underrepresented in cross-sectional food bank usage estimates compared to their proportion in the Canadian population (Food Banks Canada 2016); But for seniors who do draw on food banks, they could face intersecting challenges from fixed incomes but rising food, shelter and medical costs.

Given the lack of available data or analyses on the patterns of food bank usage, there is little current evidence about whether charitable food programs are actually used as “emergency” or short-term services versus longer-term supports. The main objective of this study was therefore to use an administrative dataset of food bank member usage from a large urban food bank organization to provide a descriptive profile of patterns of food bank usage over 25 years including estimates of the frequency and duration of use. The secondary objective was to explore individual-level predictors of duration and frequency of engagement with food banks drawn from socio-demographic characteristics reported by food bank members to assess potential factors that could reinforce longer engagement or facilitate transitioning out of the food bank system.

## Methods

### Data Source

**Fig. 1:**
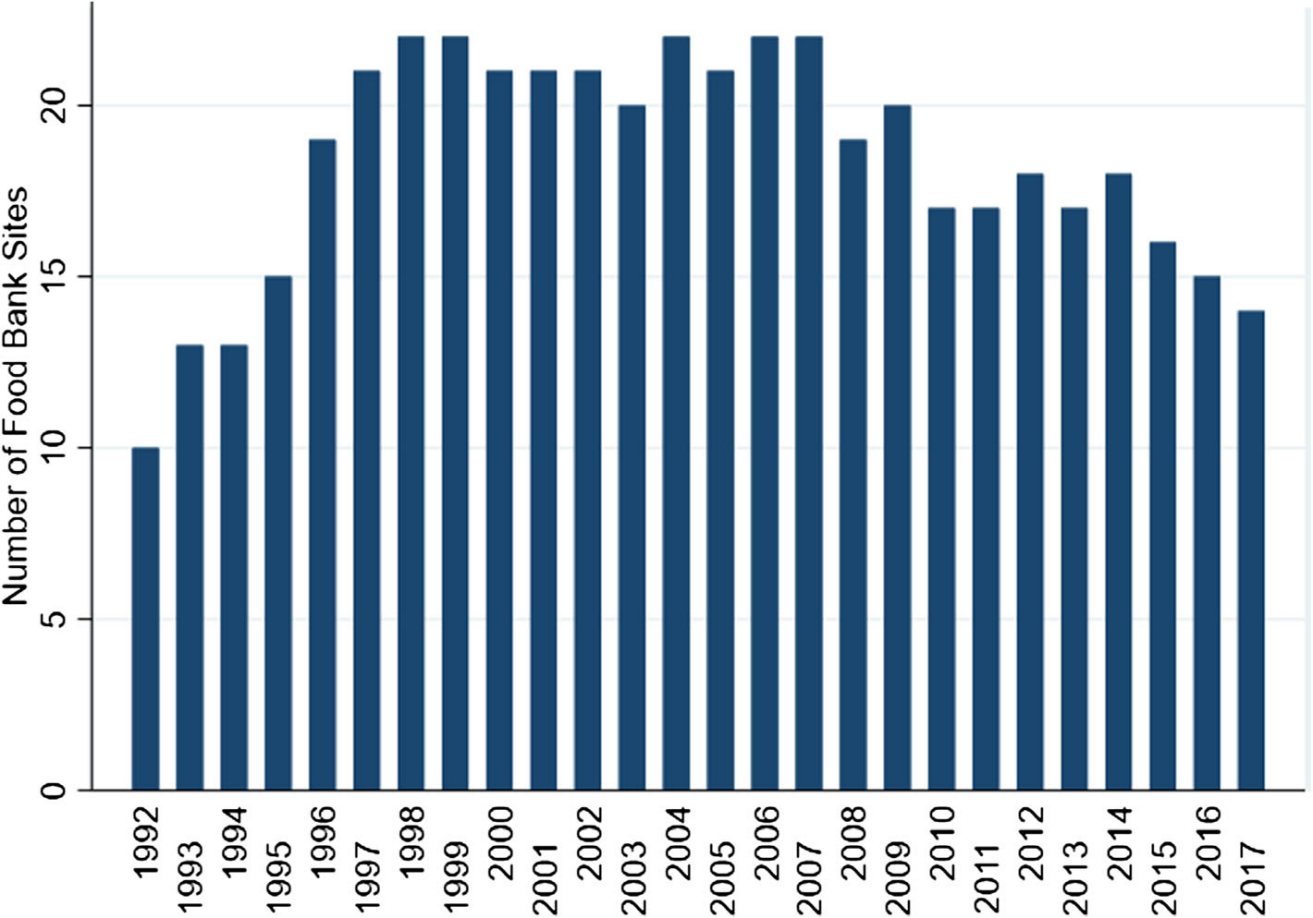
Number of food bank sites operated each year by the Greater Vancouver Food Bank (1992–2017)

This study was completed under the auspices of an ongoing research partnership with the Greater Vancouver Food Bank (GVFB), a large food banking organization in the lower mainland of Vancouver, which operates food bank sites in four municipalities (Burnaby, North Vancouver, New Westminster, and Vancouver). GVFB initiated this partnership to collaboratively address the seeming paucity of information and create a critical lens around the intended aims and impacts of food banks on food insecurity and the lived experiences of individuals served. At the GVFB, there are two main streams of food distribution: directly, through a set of food programs operated by the organization, and indirectly, where food is provided to an intermediary organization (i.e. shelters and low-barrier housing, schools, and community centres) to support their independently operated programs. With direct food provision, which is the focus of this paper, individuals register for service and are thereafter considered “members” of the organization. Drawing from the organization’s full database of member visits via direct service, this research incorporates information from January 1992 through June 2017. Over this time, data were collected about visits from 30 food bank programs, of which 14 were active in 2017 (see Fig. 1). The majority of these programs have historically offered food distribution services once per week (typically lasting from 1 to 3 h long). Most sites close 1 week each month coinciding with the distribution of social assistance cheques in British Columbia. Members are typically limited to a maximum of one visit each week but may visit any site in the organization that they choose on a given week.

Data were collected upon registration with the food bank, at which time members were asked to report their gender, birthdate, principal source of income, and the gender and birthdate of other members of their household with whom they share food bank allotments, if any. Members were not systematically required to provide updated data after initial registration, although some members updated information on a voluntary basis. Other key data collected included whether members received a designation indicating a mobility challenge which is allocated if a member provided evidence (typically a medical note or evidence of using a wheelchair) demonstrating a challenge preventing them from standing or waiting in line (as is often required when waiting for food bank services). GVFB’s database coded each member with a unique numeric identification number linked to the given member’s demographic data as well as information regarding associated visits to any of the 30 food bank programs run directly by GVFB. Visit data included the date and location of each visit for each member, which, prior to 2012, was captured in written logs on site and transferred manually to the central database. In 2012, GVFB phased in an electronic visit tracking system whereby members swipe their member identification cards before entry at a food bank program and visits are automatically added to the database.

### Measures

#### Food Bank Usage and Outcome Variables

Since there are no standard metrics for assessing food bank usage patterns, we derived a suite of variables constructed from archival logs of members’ visits to capture elements of service usage including measures of frequency, duration, and consistency of use (described further in Tables 2, 3). The elapsed time between the first and last recorded visit estimated the duration of engagement with the organization. We explored a variety of cut-offs to examine shorter versus longer engagement, and present findings (Table 3) examining engagement in the food bank system for a relatively shorter duration (defined as < 1 vs. ≥ 1 year), and the likelihood of staying engaged for a relatively longer-term duration (defined as < 5 vs. ≥ 5 years). The total number of visits on record per member captured total visit frequency and overall exposure. “Intensity” of engagement was constructed based on the number of total visits logged per member divided by number of years elapsed between first and last recorded visit. The number of unique program sites visited examined the mobility of members within the GVFB system and across sites. Episodic usage was assessed as the frequency of returning for a subsequent visit after pauses/absences of visits using a variety of pause lengths (30 days, 90 days, 1, 2, and 5 year pauses between visit dates) to examine cycles of returning after shorter and longer absences from usage.

#### Independent Variables Associated with Food Bank Usage

We focussed on socio-demographic variables which were collected regularly at the time of member registration for all years of analysis for which there was a plausible theoretical rationale for exploration and when possible could be compared to national estimates from the Hunger Count (Food Banks Canada 2016). In particular, demographic variables included: gender (coded as male, female, and unknown gender), age (in years), and age group as an ordinal variable (17–34, 35–54, 55–64, and ≥ 65 years) defined at the time of the member’s first visit. Members do not report amount of income earned but are voluntarily asked to identify principal source of income earned at time of registration, and verbal responses were coded by GVFB staff or volunteers into one of a possible 13 response options (full-time employment, part-time employment, Employment Insurance, social assistance, disability, pension, savings, immigration, other income, none, Workers’ Compensation, parental leave, or student loan). We recoded this variable aiming to develop meaningful groups for comparison including: (1) sources indicative of recent income earned though employment including part-time or full-time employment, and Employment Insurance which is available only to those who recently lost their jobs and are actively looking for work (Government of Canada 2017a), Workers’ Compensation (available to workers injured on the job) (Government of Canada 2017b), parental leave (Government of Canada 2017c), or student loans; (2) social assistance; (3) disability-related assistance; (4) pension; (5) other sources of income including savings or self-identified “immigrant” sources or no primary source; and (6) missing income source. As a proxy for substantive health or mobility challenges, we used GVFB’s records noting a presence of a mobility issue (yes/no). We note that this variable was not part of the standard member registration process and is only added to a member’s file if they sought and were approved for this designation.

We further estimated the number of members sharing food bank allotments with the “primary member”. This variable is a proxy for household size as it assesses the number of family members (or roommates, caregivers, or others living in the household) with whom the member shares food resources. We also estimated the number of children age 18 or younger who shared allotments at the time of the member’s first visit. As a proxy for single parenthood, we identified members who shared allotments with members age 18 years or younger at the year of their first visit, but who did not share with any other adults.

### Statistical Analyses

All analyses were conducted using Stata version 15.0 (StataCorp, College Station, Texas). Data were carefully inspected and cleaned to remove data entry errors (including implausible values, “test” entries used for administrative purposes, and erroneous duplicate entries). Data entries for which the member identification number was missing or erroneous were dropped. Descriptive statistics including means, medians, and standard deviations for continuous variables and frequencies and proportions for categorical variables were used to examine demographic characteristics and key food bank usage variables (see Tables 1, 2).

**Table 1:** Socio-demographic characteristics of Greater Vancouver Food Bank members by service usage cluster type (1992–2017)

Characteristic	Total population *n*/%		Cluster 1 Transitional users *n*/%		Cluster 2 Episodic users *n*/%		Cluster 3 Chronic users *n*/%	
*N*	116,963	100%	106,921	91.41%	8247	7.05%	1795	1.53%
*Gender*								
Female	49,178	42.05%	44,243	41.38%	3993	48.42%	942	52.48%
Male	66,975	57.26%	61,897	57.26%	4226	51.24%	852	47.47%
Unknown	810	0.69%	781	0.73%	28	0.34%	1	0.06%
*Age at first visit (years)*	Mean 38.9	SD 14.6	Mean 38.2	SD 14.4	Mean 45.7	SD 14.4	Mean 49.4	SD 14.5
	Median 37		Median 36		Median 44		Median 48	
17–34	51,024	43.62%	48,873	45.71%	1866	22.63%	285	15.88%
35–54	48,295	41.29%	43,260	40.46%	4166	50.52%	869	48.41%
55–64	9194	7.86%	7796	7.29%	1107	13.42%	291	16.21%
> 65	6999	5.98%	5653	5.29%	1009	12.23%	337	18.77%
Unknown	1451	1.24%	1339	1.25%	99	1.20%	13	0.72%
*Main source of income*								
Recent employment/student loan^a^	8366	7.15%	7769	7.27%	487	5.91%	110	6.13%
Savings/immigrant/other/none	16,684	14.26%	15,467	14.47%	1007	12.21%	210	11.70%
Social assistance	52,214	44.64%	48,853	45.69%	2745	33.28%	616	34.32%
Disability income	8607	7.36%	7066	6.61%	1288	15.62%	253	14.09%
Pension	3573	3.05%	3361	2.87%	584	7.08%	242	13.48%
Missing income data	27,519	23.53%	27,019	23.40%	2136	25.90%	364	20.28%
*City of residence*								
Vancouver	82,427	70.47%	75,420	70.54%	5769	69.95%	1238	68.97%
Burnaby	17,260	14.76%	15,692	14.68%	1312	15.91%	256	14.26%
North Vancouver	4705	4.02%	4372	4.09%	263	3.19%	70	3.90%
New Westminster or other	12,571	10.75%	11,437	10.70%	903	10.95%	231	12.87%
*Number of sharing members*								
0	92,774	79.32%	86,858	81.24%	4945	59.96%	971	54.09%
1	11,990	10.25%	10,098	9,44%	1494	18.12%	398	22.17%
2	6183	5.29%	5261	4.92%	746	9.05%	176	9.81%
3 or more	6016	5.14%	4704	4.39%	1062	12.87%	250	13.93%
*Household composition*^*b*^								
Shares food with 1 or more children < 18 years	15,396	13.16%	13,031	12.19%	1936	23.48%	429	23.90%
Single parent	7658	6.55%	6740	6.30%	765	9.28%	153	8.52%
*Existing mobility challenges (yes)*	1219	1.04%	532	0.50%	428	5.19%	259	14.43%

^a^This income category includes members with reported income sources from part-time or full-time employment, student loans, Employment Insurance, parental leave, or Workers’ Compensation

^b^Single parents defined as members who shared allotments with at least one person age 18 or younger the year of their first visit, but did not share with any other adults. These members would also be included in the variable “Shares food with 1 or more children < 18 years” which also includes households sharing with other adults

**Table 2:** Characteristics of food bank use by Greater Vancouver Food Bank members by service usage cluster type (1992–2017)

	Total population *n*/%		Cluster 1 Transitional users *n*/%		Cluster 2 Episodic users *n*/%		Cluster 3 Chronic users *n*/%	
*Total elapsed time (years)*	Mean 2.5	SD 4.5	Mean 1.9	SD 3.8	Mean 8.6	SD 5.4	Mean 13.8	SD 4.6
	Median 0.3		Median 0.2		Median 7		Median 13.4	
< 1 Year	73,274	62.7%	73,274	68.5%	–	–	–	–
1–5 Years	22,806	19.5%	20,139	18.8%	2667	32.3%	–	–
> 5 Years	20,883	17.9%	13,508	12.6%	5580	67.7%	1795	100%
*Total visits on record*	Mean 19	SD 50	Mean 7	SD 11	Mean 109	SD 42	Mean 322	SD 94
	Median 3		Median 3		Median 98		Median 294	
1	32,980	28.2%	32,980	30.8%	–	–	–	–
2–5	40,819	34.9%	40,819	38.2%	–	–	–	–
6–10	12,448	10.6%	12,448	11.6%	–	–	–	–
11–50	19,392	16.6%	19,392	18.2%	–	–	–	–
> 50	11,324	9.5%	1282	1.2%	8247	100%	1795	100%
*Visits per year*	Mean 15	SD 31	Mean 15	SD 33	Mean 17	SD 10	Mean 25	SD 7
	Median 5		Median 3		Median 16		Median 26	
*Number of unique sites visited*	Mean 2	SD 1	Mean 2	SD 1	Mean 3	SD 2	Mean 3	SD 2
	Median 1		Median 1		Median 2		Median 3	
1	76,982	65.8%	73,818	69.0%	2669	32.36%	495	27.6%
2	23,066	19.7%	20,696	19.4%	1997	24.21%	373	20.8%
3 or more	16,915	14.5%	12,407	11.6%	3581	43.4%	927	51.7%
*Episodic use*								
*30 day pauses*	Mean 3	SD 5	Mean 2	SD 3	Mean 11	SD 9	Mean 14	SD 10
	Median 1		Median 1		Median 9		Median 12	
1 30-day pause	21,248	18.2%	20,819	19.5%	378	4.6%	51	2.8%
2–5 30-day pauses	26,392	22.6%	24,106	22.6%	1955	23.7%	331	18.4%
> 5 30-day pauses	16,312	14.0%	9225	8.6%	5700	69.1%	1387	77.3%
*1 year pauses*	Mean 0	SD 1	Mean 0	SD 1	Mean 1	SD 1	Mean 1	SD 1
	Median 0		Median 0		Median 1		Median 0	
1 full-year pause	19,112	16.3%	16,464	15.4%	2169	26.3%	479	26.7%
2–5 full-year pauses	11,028	9.4%	8472	7.9%	2201	26.7%	355	19.8%
> 5 full-year pause	270	0.7%	176	0.2%	90	1.1%	1	0.1%
*5 year pauses*	Mean 0	SD 0	Mean 0	SD 0	Mean 0	SD 0	Mean 0	SD 0
	Median 0		Median 0		Median 0		Median 0	
1 or more 5 year pauses	9736	8.3%	8190	7.7%	1361	16.5%	185	10.3%

Cluster analysis using the k-means function in Stata was applied to construct unique clusters to describe robust divisions between groups of food bank members that may have been missed using exploratory and descriptive statistics alone. The findings present a three-cluster solution based on a simplified set of usage characteristics derived from: total number of food bank visits, total time in years elapsed between first and last recorded visit, the number of 90 day pauses between visits (as a proxy for episodic use), and the number of unique sites visited (as a measure of intra-organizational mobility). The naming of clusters was informed by the resulting findings, but also aligned with typologies identified regarding patterns of shelter utilization among homeless adults (Kuhn and Culhane 1998; Aubry et al. 2013). Further analyses then explored whether socio-demographic characteristics differed by usage clusters (Table 1).

To examine the robustness of findings from cluster analyses, several regression analyses were conducted to explore predictors of usage duration and frequency, and likelihood of disengaging with the food bank (see Table 3). First, linear regression analyses examined predictors of total elapsed years between first and last visit (column II), and Poisson regression estimated predictors of the number of total visits made to the food bank (column III). The robustness of findings from these two modelling approaches was examined with logistic regression approaches with dependent variables defined as: having elapsed time between first and last visit of equal to or greater than 1 year (vs. less than 1 year) (column IV), and having an elapsed time between first and last visit of equal to or greater than 5 years (vs. less than 5 years) (column V). Cox proportional hazard models further examined associations between independent variables with the elapsed time before disengaging from food bank use (defined here as the last date a food bank program was visited on record) (column VI). To interpret results from hazard models, values above 1 signify an increased risk (higher hazard) of *disengaging* from the food bank, whereas an estimate below 1 indicates a lower risk of disengaging (and hence a higher risk of remaining engaged and returning for a subsequent visit) compared to the reference category, controlling for all other variables in the model.

**Table 3:** Results of adjusted regression models and 95% confidence intervals examining associations with elapsed years of food bank engagement, number of total food bank visits, odds of remaining engaged after 1 year and 5 years, and cumulative hazards of engagement

	Elapsed years^a^	Total visits^b^	Returned after 1 year^c^	Returned after 5 years^d^	Hazard model^e^
*Gender*					
Female (reference)	0 [0, 0]	1 [1, 1]	1 [1, 1]	1 [1, 1]	1 [1, 1]
Male	0.24*** [0.18, 0.29]	0.98 [0.95, 1.01]	1.12*** [1.08, 1.15]	1.16*** [1.12, 1.20]	0.93*** [0.92, 0.95]
Unknown gender	− 1.01*** [− 1.22, − 0.80]	0.31*** [0.24, 0.40]	0.38*** [0.30, 0.49]	0.20*** [0.12, 0.33]	1.14* [1.03, 1.27]
*Age at first visit (years)*	0.002*** [0.0002, 0.003]	1.02*** [1.02, 1.02]	1.01*** [1.02, 1.01]	1.004*** [1.002, 1.005]	0.997*** [0.996, 0.998]
*Primary income source*					
Recent employment/student loan^e^ (reference)	0 [0, 0]	1 [1, 1]	1 [1, 1]	1 [1, 1]	1 [1, 1]
Savings/immigrant/other income/none	− 0.37*** [− 0.48, − 0.27]	0.99 [0.93, 1.06]	0.82*** [0.77, 0.87]	0.80*** [0.73, 0.86]	1.15*** [1.10, 1.19]
Social assistance	− 0.24*** [− 0.34, − 0.14]	0.98 [0.93, 1.05]	1.08** [1.02, 1.14]	0.96 [0.90, 1.03]	1.04* [1.11, 1.19]
Disability income	3.15*** [3.00, 3.30]	1.82*** [1.69, 1.95]	3.65*** [3.40, 3.92]	4.25*** [3.62, 4.61]	0.60*** [0.58, 0.62]
Pension	1.28*** [1.10, 1.46]	1.57*** [1.44, 1.70]	1.70*** [1.55, 1.86]	2.14*** [1.92, 2.39]	0.85*** [0.81, 0.88]
Unknown income	1.61*** [1.48, 1.73]	1.88*** [1.75, 2.02]	1.91*** [1.78, 2.04]	2.69*** [2.46, 2.94]	0.78*** [0.76, 0.81]
*Number of sharing members*	0.80*** [0.77, 0.83]	1.33*** [1.29, 1.33]	1.60*** [1.57, 1.63]	1.61*** [1.58, 1.64]	0.85*** [0.84, 0.85]
*Known mobility challenge (yes)*	6.60*** [6.26, 6.94]	3.85*** [3.58, 4.14]	15.76*** [13.10, 18.95]	32.39*** [26.37, 39.79]	0.42*** [0.40, 0.43]
*Single parent*	0.26*** [0.16, 0.37]	1.16*** [1.10, 1.22]	1.12*** [1.06, 1.19]	1.26*** [1.17, 1.40]	0.94*** [0.92, 0.96]
*N*^f^	107,826	107,826	106,614	97,602	75,340

All models additionally controlled for year of member’s first visit, city of residence (Vancouver, Burnaby, New Westminster, North Vancouver, or unknown/other), and an indicator variable for the food bank program site most frequently visited. All models used robust standard errors

^a^Ordinary least squares regression (beta coefficients)

^b^Poisson regression (incidence rate ratios)

^c^Logistic regression (odds ratios)

^d^Non-censored Cox proportional hazards models (hazard ratios)

^e^The reference group includes members who reported income from part-time or full-time employment, student loans, Employment Insurance, parental leave, or Workers’ Compensation

^f^9137 were dropped from fully adjusted regression analyses owing to missing data on variables included in the models; logistic regression models of odds of returning after 1 or 5 years excluded members whose date of first visit was less than 1 or 5 years, respectively, before June 2017 and hazard models excluded members who only came for a single visit and never returned

For all modelling approaches, first a set of exploratory univariate analyses examined associations with the following independent variables: gender, age, main income source, number of members sharing food bank allotments, single parenthood (yes/no), and the presence of a noted mobility issue (yes/no). We were interested in examining the associations between key independent variables after accounting for several potentially confounding effects that could alter food bank usage patterns. We did not have access to members’ specific residential addresses with which to evaluate impacts of proximity to food bank locations on usage, nor knowledge of their housing type, residential stability or ethnic or cultural background. However, we controlled for reported city of residence at the time of registration which could impact members access to physical locations and transportation networks. We also controlled for the food bank site most frequently visited to account for potential differences in service availability and quality across sites, and for the year of the members’ first visit to account for potentially confounding characteristics associated with year of onset of engagement. A final set of fully adjusted models is reported in Table 3, and robust standard errors were used in all models.

## Results

### Population Characteristics

Between January 1992 and June 2017, at least 116,963 primary member identification numbers were issued and recorded by GVFB, with over 2 million unique visits logged by these members. As shown in Table 1, there were more male (57%) than female (42%) members, and mean age at first visit was 39 years old (SD = 15). Forty-five per cent of members reported social assistance as their main source of income, 8% reported no income at all, and 7% reported earning disability-related income, while 7% (combined) reported income from full-time or part-time work or Employment Insurance and 3% relied on income from a pension. Note that members’ reports of disability income did not differentiate between income provided by provincial social assistance versus federal or private pensions related to disability. Twenty-four per cent of members were missing income-related data, and the amount of missing income data grew substantially in recent years as GVFB has aimed to reduce barriers and stigma during registration including softening requirements to report income source.

**Fig. 2:**
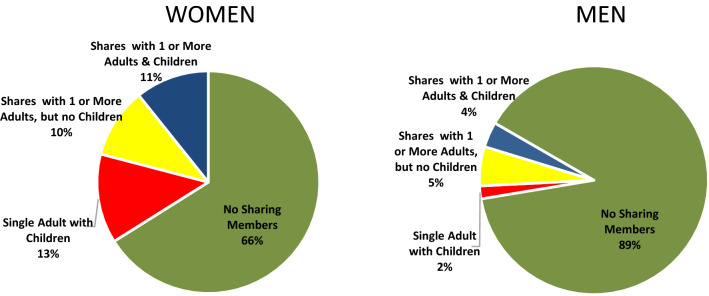
Household composition of primary food bank members. Members’ household composition was classified based on the number of sharing members on record and the age of the sharing members at the year of the primary member’s first visit

Most members (79%) did not share their food bank allotments with other household members, while 10% shared with one and 10% shared with two or more members. Among members who did share their allotments with others, 72% shared with at least one child age 18 years or younger at the time of the primary member’s first food bank visit and 7% of all members shared food with another child, but no other adults (used here as an indication of likely single parent households). A higher proportion of women (13%) shared allotments with children under age 18 but no other adults versus men (2%), and the majority of men (89%) did not to share with others (vs. 66% for women) (Fig. 2). The majority of members lived in the city of Vancouver (70%) where most GVFB food bank sites are located, followed by Burnaby (15%), New Westminster (8%), or North Vancouver (4%), all of which currently house one GVFB site. Two per cent of members had missing data about city of residence, and less than 1% reported residing in other regions in British Columbia or in rare cases, outside the province. One per cent of members were designated as having pre-existing mobility issues.

### Patterns of Food Bank Usage

Since 1992, 30 distinct food bank site locations have been in operation including an annual “grocery bag” pickup program, wherein once per year, members can access an extra hamper of foods during business hours at the GVFB main warehouse which also houses their administrative offices. Usage levels varied widely by site, with the two smallest (and shortest lived) sites logging less than 2400 member visits, while the single most frequented program amassed 265,130 visits (comprising 12% of all visits on record). Nine sites logged over 100,000 visits each and collectively provided 69% of all member visits. Annual grocery bags made up only 3% of all visits, but more than 1 in 4 members (27%) made use of the grocery bag program at least once, and it served as the first program accessed for 18% of members.

**Fig. 3:**
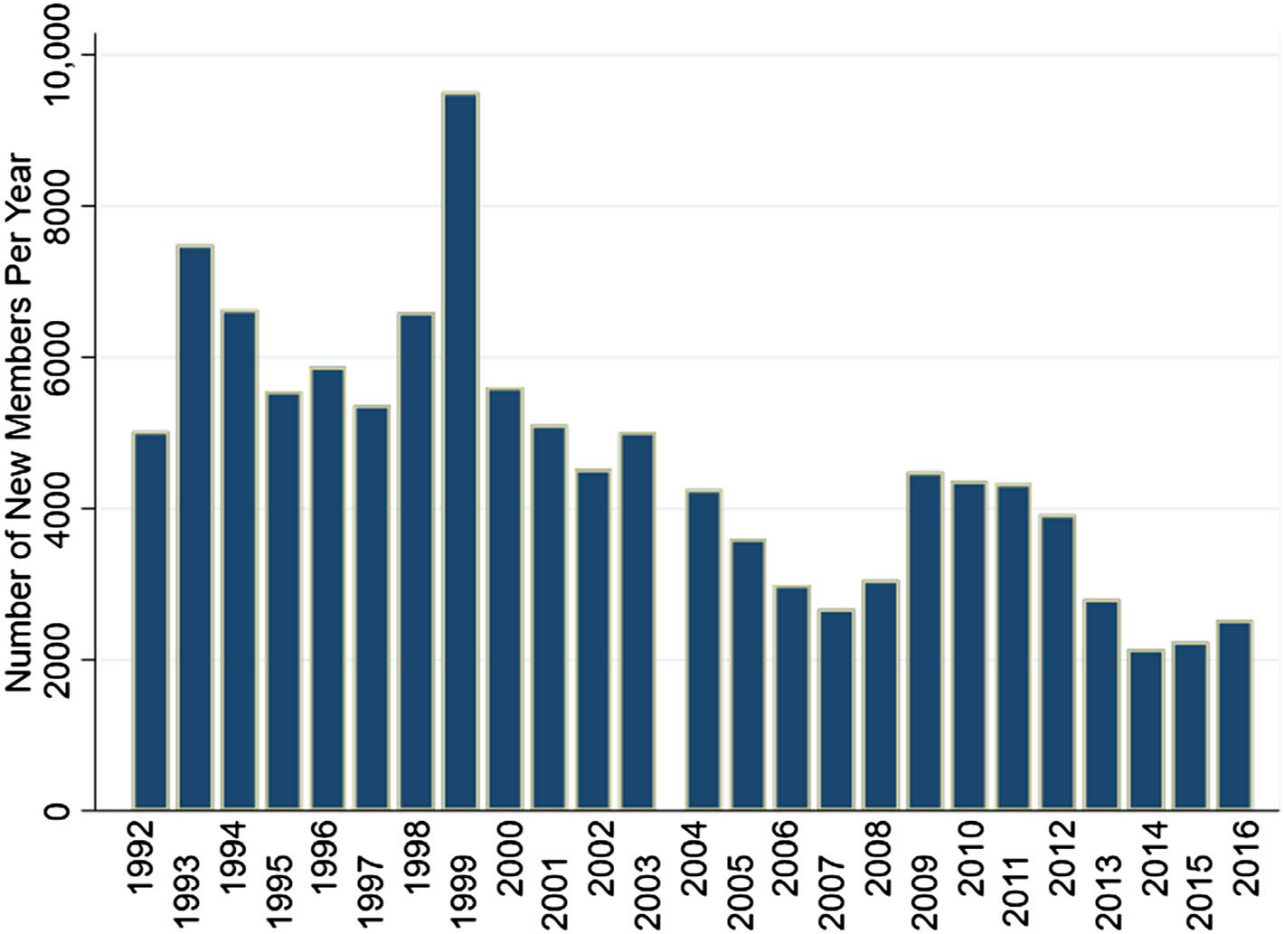
Number of new members logging a first visit each year at the Greater Vancouver Food Bank (1992–2016). This graph excluded 2017 because data were only available until June of that year

Figure 1 describes the patterns of change in availability of GVFB food banks between 1992 and 2017. The number of locations grew rapidly in the early 1990s doubling from 10 sites in 1992 to peak of 22 sites in 1999. The number of new GVFB members also varied over time (mean new members per year = 4625, SD = 1765), with a peak of 9504 new members in 1999 (Fig. 3). There was also a modest increase in annual visits and new members following the 2008 economic recession that returned to below pre-recession levels by 2014 (Fig. 3).

Overall, twenty-eight per cent of members came for a single visit and never had a second documented visit (Table 2). While most members returned to the organization after their first visit, the majority of members (74%) logged ten or fewer recorded visits. The distribution of usage frequency was highly skewed with median total visits of 3, but mean total visits approximating 20 (SD = 50) and clear differences between longer-term and shorter-term members in number of visits, frequency, and intensity of use. Members within the top 5th percentile of usage, had 99 or more visits, and over 2000 individuals had 200 or more visits. The majority (63%) of members came for food bank visits over the course of less than 1 year (estimated by the elapsed time between their first and final visits on record), while 18% of members were engaged with the food bank for over 5 years. Those at or above the 95th percentile of elapsed time were engaged for over 13 years, with over 1000 members on record as being engaged for over 20 years. Still, over half of the members engaged for over 20 years, visited 20 times or less, suggesting diverse patterns of use even among the longest-term members (see also Fig. 5).

Intermittent food bank use was common. That is, members frequently took pauses between use, rather than maintaining continuous (weekly), ongoing use. Among members who were engaged with the food bank for 1 year or more, 99% had at least one 30-day pause in use (where no visits were logged for at least 30 days in a row) and half of these members had 4 or more episodes of pausing for at least 30 days and then returning. Among members engaged with GVFB for longer than 1 year, 70% had at least one full-year pause between visits. For long-term members engaged with GVFB for 5 years or more, 49% had 2 or more full-year pauses between visits. Intensity of use (measured here as the number of visits divided by the number of years elapsed between first and last recorded visit) was highly skewed and varied widely, with median intensity equal to 5 visits per year, but mean of 15 (SD = 31). Most members (66%) only ever visited one food bank site, while those in the highest 5 percentiles of site number usage visited 4 or more unique locations.

### Typologies of Food Bank Usage

**Fig. 4:**
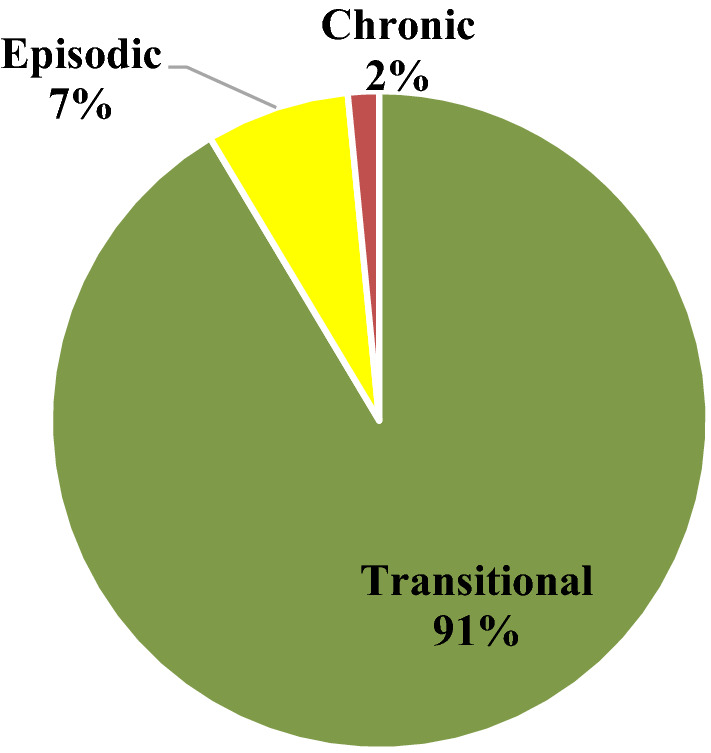
Proportion of food bank members, by service usage cluster type

**Fig. 5:**
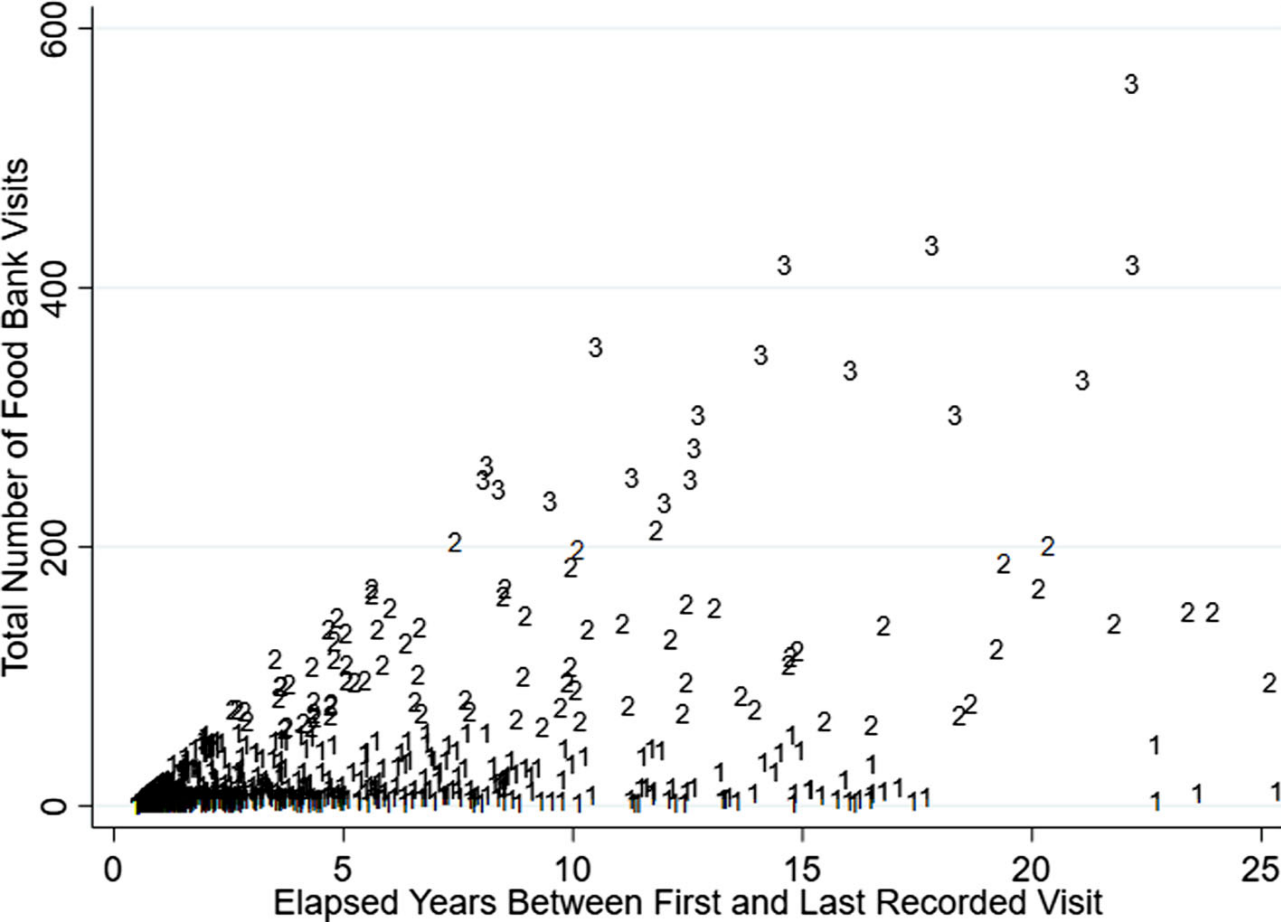
Elapsed years between first and last visit and total number of food bank visits, by cluster group for a random sample of food bank members. This graphic represents a stratified random sample of 1% of each cluster subgroup (*n* = 1169). 1 = transitional members (*n* = 1069); 2 = episodic members (*n* = 82); 3 = chronic members (*n* = 18)

**Fig. 6:**
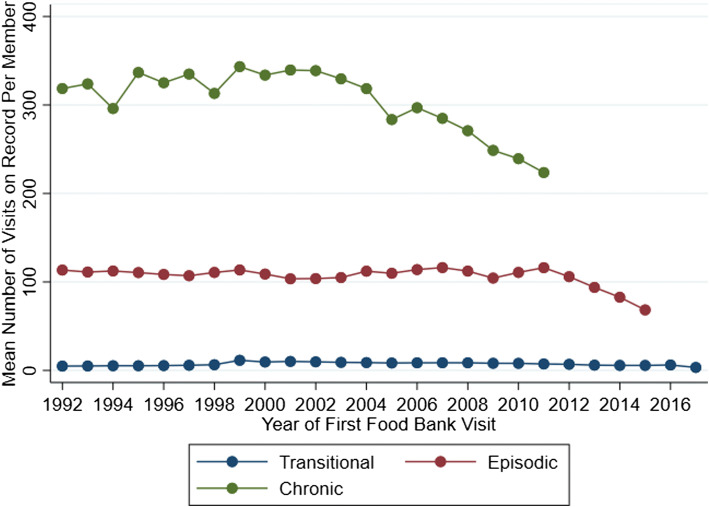
Mean number of visits on record per member by cluster grouping, by year of members’ first visit

Cluster analysis revealed three distinct typologies of food bank use characterized here as short-term or “transitional” use, medium-term or “episodic” use, and longer-term “chronic” usage (see Fig. 4 and Table 2). The majority (91%) of food bank members represented transitional users who engaged for a relatively short bout of time (median elapsed time for this cluster = 62 days), and came for a relatively small number of food bank visits (median = 3, mean = 7). Figure 5 further shows a graphic representation of a stratified random sample of 1% of each cluster group to depict the distribution of total visits and elapsed years of food bank usage across these three clusters. As described in Table 1, transitional users were more likely to be male (57%), were least likely to share their food bank allotments with other members or with children (81% had no sharing members and 12% shared with one or more children), and were slightly younger on average at the time of their first visit (mean age first visit = 38, median = 36 years) compared to the full population. Figure 6 shows that the mean number of visits among transitional members remained relatively stable over time (regardless of the year that the member first engaged with GVFB) and highlights the large differences in the mean number of total visits amassed between members based on their cluster type.

Seven per cent of members (*n* = 8247) were characterized as medium-term, episodic service users. This group’s use was not short term or fleeting, with mean elapsed time engaged of 8.6 years (median = 7 years), with mean number of visits over 100 (median = 98). This cluster had the highest average number of usage pauses that lasted 90 days or longer between visits, highlighting the episodic nature of the usage (mean number of 90 day or longer pauses = 4).

A small group of 1795 members (1.5%) exhibited patterns of chronic food bank use. These members visited the food bank at least 200 hundred times each (mean = 322, median = 294) over several years (mean and median > 13 years, minimum = 6 years) with mean visit intensity of 25 visits per year. Figure 6 shows that the mean total amassed visits among chronic service users varied depending on the year of first engagement in the food bank but remained far higher in all years than episodic or transitional users. Moreover, the estimates for total visits are still subject to rise as many of the chronic members in Fig. 6 will continue to make visits after 2017. Chronic service users were slightly more likely to be female (52%) and were on average 11 years older at the time of their first visit (mean age = 49, median = 48 years) compared to the general population of members. These members were more likely to share their hampers with other household members and with children (46% shared and 24% shared with one or more children). Compared to transitional members, episodic and chronic service users were more likely to rely on disability income and pensions and had far higher proportions of members with pre-existing mobility challenges (e.g. 14% among chronic members vs. 0.5% for transitional). Episodic and chronic members also had slightly higher proportions of members who shared food with children but no other adults.

### Predictors of Food Bank Usage

**Fig. 7:**
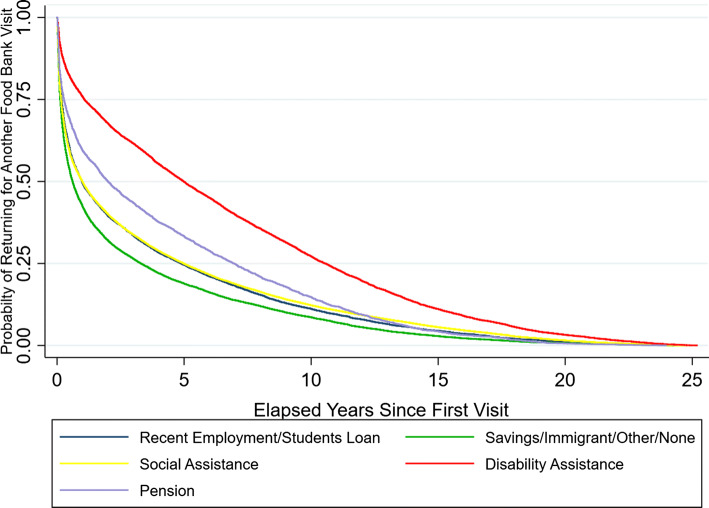
Kaplan–Meier survival curves by primary income source. Probability estimates displayed on the *y*-axis describe the proportion of primary food bank members who returned for a subsequent visit beyond the time specified on the *x*-axis

Regression analyses shown in Table 3 confirmed several key findings that emerged from descriptive and cluster analyses including strong and significant associations between duration and frequency of food bank engagement with income source, reported mobility challenges, and reliance of other household members on food bank allotments, and a small but positive association between increased age and food bank engagement. Fully adjusted models found that compared to members with income sources related to recent employment or student loans, members relying on disability benefits and pensions were far more likely to remain engaged with the food bank, were engaged for a longer time (3.2 and 1.3 years longer, respectively) and netted more total visits. (Incident rate ratios (IRR) for total visits were 1.8 and 1.6, respectively.) In contrast, members who reported drawing primarily on personal savings, income following immigration, other or no income sources were 15% more likely to *disengage* [hazard rate (HR) = 1.15] and had shorter elapsed usage times compared to the reference group. Figure 7 depicts Kaplan–Meier survival curves based on members’ primary source of income. These curves show that members reporting disability assistance maintained the highest probability of returning for more food bank visits over time (red line), followed by those with pension income (purple line), while those reporting income from savings, immigrant, other or none had the lowest probability of returning for another visit.

Moreover, those with reported mobility issues stayed engaged for over 6 years longer than members without noted mobility challenges, and accrued more than three times as many total visits (IRR = 3.9). Members with mobility challenges also had substantially lower hazard ratios (HR = 0.42) indicating a 58% lower risk of disengaging from the food bank compared to members without mobility challenges, higher odds of staying for over 1 year (OR = 15.8) and were 32 times more likely to remain engaged after 5 years, with robust results across models. It should also be noted that program-level policy, by design, allows mobility-designated members priority service access.

Having other members of the household reliant of food bank allotments was also a significant predictor of increased usage. Each additional household member sharing a member’s food supplement was associated with approximately 10 months of increased elapsed food bank engagement and 33% more total visits (IRR = 1.33). Across all models, number of sharing members remained a significant predictor of increased usage and lower likelihood of disengagement. Even after controlling for the number sharing of members and other covariates, members sharing allotments with children but no other adults (a proxy for single parent households) were more likely to stay engaged and report more visits than members who were not living in single parent households.

Increased age was associated with a small but positive relation with increased food bank engagement across models. For example, an increase in 1 year of age was associated with a 2% higher rate of total visits (IRR = 1.02). Gender was significantly associated with food bank usage in some models, but statistical significance, direction, and magnitude of associations were generally small, inconsistent, and sensitive to model specification and type of usage outcome examined. For example, while men had slightly higher elapsed time engaged and were less likely to disengage compared to women in the hazard rate models (HR = 0.93), no differences were found in total number of visits between men and women in adjusted models.

While this analysis was interested in the pooled results from across the food bank programs in the GVFB organization, patterns of use differed between food bank program sites, depending on city of residence and year of first visit. While it is beyond the scope of this analysis to examine geographic differences in specific site or related social policy changes that shaped usage patterns, the potentially confounding effects of site, city of residence, and other unmeasured characteristics associated with commencing use in a given year were controlled for in adjusted models. It is also worth noting the substantial interconnectedness between food bank usage outcome variables. For example, visiting more than one site was associated with substantially higher engagement across models, and there were significant positive linear associations between the numbers of unique sites visited, total number of visits, elapsed years engaged, and number of pauses or episodes of usage.

## Discussion

This study drew on a rich source of data that tracked detailed patterns of food bank use among a population of over 100,000 individuals for 25 years from one of Canada’s largest food bank organizations. To our knowledge, this is the first analysis of its kind in North America, providing valuable insight into the varied patterns of engagement used by individuals that draw on food bank programs. Findings suggest that overall, the majority of members from this Vancouver-area food bank organization can be characterized as short-term, transitional users who visited food banks a handful of times and disengaged after a few weeks or months of use. On the other hand, food banks also clearly provide ongoing supports to a small subset of longer-term users, some of whom cycle in and out of the food bank system, while others remain deeply engaged and come regularly over the course of many years and in some cases decades. While episodic and chronic users collectively comprised only 9% of the food bank population, they accounted for 65% of all food bank visits. This reveals that a substantial proportion of food bank resources are providing ongoing food supports beyond what can be considered an acute or emergency context.

To our knowledge, there are no other long-term longitudinal data from other food bank organizations with which to compare these findings. However, a recent British study using data from the West Cheshire food bank similarly found that between 2013 and 2015, 55% of households came for only a single visit, but that the 16% of users with the highest frequency of visits (4 or more visits between 2013 and 2015), comprised 43% of all visits (Garratt 2017). In a small (*n* = 270) cross-sectional convenience sample of food bank users in the boroughs of London, England, 49% and 30% of study participants reported coming only once or twice to the food bank in the past 6 months, respectively, and only 9% visited four or more times (Prayogo et al. 2017). On the other hand, a recent report of food bank users from three German cities found that the majority of users (70%) were engaged for over 12 months with most (68%) reporting visiting 4 or more times each month (Depa et al. 2018), suggesting that service models and usage patterns vary greatly in different contexts.

Findings here also point to several life circumstances that were strongly associated with deeper, longer engagement with food bank services. In particular, members relying on pensions or disability-related assistance as their main sources of income (compared with members recently earning income or using student loans), and members with disclosed health and mobility challenges, were consistently and substantially more likely to have higher engagement. Directions of these findings were consistent regardless of how engagement was modelled and results remained robust after controlling for a wide set of other potentially confounding factors.

Current findings hence align with growing evidence in Canada and internationally that food bank use is deeply connected to extreme financial vulnerability, and experiences of disability and state welfare reforms (Garthwaite et al. 2015; Holmes 2017; Loopstra and Lalor 2017). Indeed, the provincial welfare benefit system has been critiqued for being structurally dependent on food banks and other charities to assist people in meeting basic needs in the wake of neoliberal policy changes (McBride and McNutt 2007). In British Columbia specifically, the welfare system caseloads and proportions of forms of assistance received have changed substantially in the past 25 years. For example, Pulkingham notes a steep drop in the rates of entrance to social assistance reported from the mid-1990s through the rest of that decade (Pulkingham 2015). While the current study did not formally test the associations between welfare policies and food bank use, the late 1990s was also a time of substantial growth in the number of GVFB food banks, rates of new members joining, and growth in annual visits to the organization.

There is also evidence and reports from social services staff and recipients that provincial government staff divert applicants for social assistance to food banks and emergency shelters (Wallace and Klein 2006). Even among those able to access social assistance in BC, income levels have been critiqued for being insufficient for meeting the daily food needs of households (Vu et al. 2008). For example, Klein et al. (2008) estimated that among a sample of participants who had been on social assistance for at least 15 months, 46% reported that they had often been hungry during the past month. Moreover, 77% of participants reported receiving food from a food bank, soup kitchen, or drop-in centre during the previous month, and the majority visited charitable food programs multiple times (Klein et al. 2008). Klein et al.’s 2-year longitudinal study further found that persons able to return to the labour market and leave social assistance programs voluntarily reported lower food bank usage and less severe experiences of hunger (both while on social assistance and after exiting the program).

Pulkingham (2015) further notes the trend towards a “medicalized system of income support” in British Columbia, where individuals on “temporary assistance” have declined steeply while the rates of persons reliant on disability assistance have risen (Pulkingham 2015). As of 2013, disability assistance cases made up the majority (62%) of the total welfare caseload compared to just one-tenth in 1995 (increasing steeply despite the overall decline in social assistance caseloads). Disability cases are also more likely to be on assistance for longer according to BC Government, and in 2013, nearly 90% of individuals receiving assistance for over 60 months were receiving disability assistance (Government of Canada 2016a, b). Together with the current study, these findings demonstrate the need for further investigation into the interconnected roles and impacts of provincial and federal social assistance systems on food bank usage patterns and food (in)security outcomes, particularly for persons relying on disability-related supports who may come to rely on food banks as a long-term food augmentation strategy.

This study’s findings also draw attention to the experiences of seniors who rely on the food banking system, particularly in British Columbia, a province that in 2015 reported the highest of any provincial poverty rate among seniors age 65 and over (SPARC BC 2018). While reported prevalence of food insecurity in Canada is lower among Canadians over age 65 and those receiving federal pension benefits (Emery et al. 2013; McIntyre et al. 2016), findings here suggest that older adults and those reporting pensions as their main source of income experience barriers to disengaging from food bank use. Moreover, there is evidence that both average age and the proportion of seniors engaging with GVFB are rising. In 1992, only 4% of new food bank users were 65 +, compared to 12% in 2017.

This study can also inform ongoing debates and critiques regarding the goals and activities of charitable food programs. Previous discussions have lacked empirical data to probe critical questions about whether charitable food programs are used to meet short-term, “emergency” needs, or are rather serving as a long-term resource in the absence of more effective poverty reduction strategies. Results from this study suggest that both experiences are salient. While the majority of users engaged with the food bank for only a short time, little is known about these members’ experiences or reasons for disengaging. For some, their economic or personal circumstances may have improved, reducing the need to seek supplemental foods through the charitable system. This study does provide some empirical support for this pathway given that members reporting income sources suggestive of recent employment or access to personal savings were more likely to disengage than those relying on disability assistance or pensions. However, this study cannot capture the extent to which disengagement was related to known barriers to food bank participation (including poor physical access due to transportation barriers or timing of food bank hours), or the impacts of stigmatizing or negative experiences, as examples. In a recent mixed methods study from a small sample of GVFB food banks, the majority of participants reported feeling safe and respected at Vancouver food banks (Holmes 2017). But Holmes and others have also identified several challenges in terms of service delivery including long line ups, stressful environments, and insufficient quantity and quality of food to meet needs or preferences that could also discourage repeated visits (Poppendieck 1998; Wakefield et al. 2012; Riches and Tarasuk 2014; Middleton et al. 2018).

Current findings clearly show that the lives of some food bank members are deeply connected with an ongoing relationship with food banks over many years. Although significant and ongoing financial vulnerability is a factor in long-term food bank engagement, there has been little empirical study to more deeply examine the reasons for or effects of longer-term engagement. For some, ongoing use may be shored up by the creation of social networks and community linkages that provide a space for some members to find connection and “spaces of care” (Cloke et al. 2017). For example, 4% of current members also served as food bank volunteers with evidence that volunteers had more engaged usage patterns. Longer-term members may also build relationships and supports within the food bank organization or find other incentives for participation beyond meeting household food needs.

But the presence of long-term use also likely suggests sustained barriers to meeting food needs. Given that longer-term usage was strongly connected with income source, existing mobility challenges, and an increased number of household members sharing food, single parenthood, and older age, the ability to move out of the food bank system is likely constrained by several systemic and life-course factors. In our recent work with a small convenience sample of GVFB members, we found that members reported very high rates and severity of food insecurity and health challenges. Moreover, neither length nor frequency of food bank use was associated with reduced odds of experiencing severe food insecurity (Holmes 2017), which can include reductions in food quality or quantity, experiencing hunger, weight loss, and skipping meals.

It is also worth noting that 7% of this population were characterized as episodic, longer-term users, and that both long and short duration pauses/cycles of usage were common even among shorter-term users. The evidence of episodic usage suggests that food banks may play a role for members with fluctuating economic circumstances. We unfortunately had no linked personal data about how changes in life circumstances (including access to housing or homelessness, employment or other social services or personal supports) impacted food bank patterns, and a valuable next step for research would be to examine how changing policy, funding contexts, and access to personal financial resources can improve food insecurity outcomes.

There are several limitations to keep in mind when interpreting findings from this study. First, while GVFB is a significant provider of supplemental food in the region, the study did not capture food provided through alternative programming, nor the dozens of other food banks or charitable food programs nearby. Moreover, these data are from a single organization providing service in one Canadian metropolitan area and may not reflect patterns of use from other regions or organizations. Looking into this “service ecosystem” may well yield more complex dynamics with respect to food program access patterns. Estimates reported here also likely underestimate duration and frequency of food bank usage for several reasons. First, members occasionally forget or lose their membership identification cards, and we cannot account for visits that were not logged in the administrative database. GVFB estimates that at least 1000 members have used more than one identification number (owing to lost or replaced id cards over time) and it was not possible to identify these members because data were anonymized prior to analysis to maintain confidentiality.

Metro Vancouver is among Canada’s most expensive housing markets but is also among Canada’s cities with the largest proportion of households earning low incomes (City of Vancouver 2015) which can shape both supply and demand for supplemental food. Historically, GVFB has typically restricted visits to once per week approximately 3 times each month. However, for this analysis, there were no available historical or program-specific records regarding implementation of varied visit limits or other policies with which to reflect on organizational barriers or facilitators of usage over time. For example, ease of access to information about where food bank sites are located has varied historically and is now more readily available than in early years. The organization has also changed greatly in size and staffing since its original 10 sites operated in 1992. For example, since 2012, GVFB has been under new leadership resulting in a distinct organizational shift towards more open and dynamic member engagement, aiming to design more welcoming and dignified spaces with a mission to “create empowering environments that provide and promote access to healthy food, education and training” (Greater Vancouver Food Bank 2017). Hence, it is possible the organization itself has reduced barriers to deeper engagement and has shifted the organizational culture to promote increased usage.

It is also important to acknowledge that all food bank statistics, regardless of their quality, underestimate the true prevalence of food insecurity. While Food Banks Canada estimates that under one million people in Canada used food banks over a 1 month time period in the years recently on record, national survey data estimate that over three million Canadians have inadequate or uncertain access to sufficient food because of financial constraints (Tarasuk et al. 2016). Even if questions about usage were included on nationally representative surveys (such as those integrated in the US Current Population Survey), such surveys still likely underrepresent usage owing to limitations in sampling (where low-income participants, and those with tenuous housing are less likely to participate in surveys in the first place) and estimates may also be biased by recall or social desirability biases.

Hence, this work speaks to the value of building trust and collaborations between food bank organizations and academic partners. The data collected by GVFB were the result of many years of institutional effort in attempting to create a fair and consistent approach to food distribution. In doing so, they created a remarkable archive of administrative information well suited to examining empirical questions about member usage, to inform evidence-based decision making within the organization, and potentially for extended usage in relation to social policy formulation. Strengths of this study therefore largely draw from the richness of these data amassed over 25 years that are not prone to sampling bias (since the data cover the entirely of the population) and are not subject to errors that would be inherent in trying to ascertain such data from members’ self reports of usage.

## Conclusions

This study provides insight about the patterns and predictors of food bank use over 25 years from a large Canadian urban food bank organization. Findings suggest that there are distinct typologies of food bank usage reflecting diverse experiences and roles of food banks in members’ lives. While the majority of users engaged with food bank services for a short duration with limited frequency of visits, the majority of visits overall were made by a small subset of deeply engaged longer-term members, some of whom cycled in and out of the food bank system for many years, while others had sustained engagement over many years and sometimes decades. These findings suggest the need for a more explicit dialogue among food bank leaders, supporters, and policy makers around which models of programming are being sought and the role of food banks in advocating for policy interventions that can better meet the needs of shorter- versus longer-term users. This study provokes questions about broader social policies, specifically those related to income and disability assistance programs, and supports for people with health challenges and seniors which underlay the need for and use of charitable food programs including food banks.

## References

[CR1] Aubry T (2013). Identifying the patterns of emergency shelter stays of single individuals in Canadian cities of different sizes. Housing Studies.

[CR2] Booth S, Whelan J (2014). Hungry for change: The food banking industry in Australia. British Food Journal.

[CR3] Cabili, C., Eslami, E., & Briefel, R. (2013). *White paper on the Emergency Food Assistance Program (TEFAP)*. Alexandra, VA: U.S. Department of Agriculture, Food and Nutrition Service, Office of Policy Support. https://fns-prod.azureedge.net/sites/default/files/TEFAPWhitePaper.pdf. Accessed Sept 20, 2018.

[CR4] Caraher, M., & Furey, S. (2018a). The growing problems of food poverty and insecurity. In *The economics of emergency food aid provision: A financial, social and cultural perspective* (pp. 1–24). Cham: Palgrave Pivot.

[CR5] Caraher, M., & Furey, S. (2018b). The cultural and economic dimensions of food poverty. In *The economics of emergency food aid provision: A financial, social and cultural perspective* (pp. 49–72). Cham: Palgrave Pivot.

[CR6] City of Vancouver. (2015). *Social indicators and trends 2014*. http://vancouver.ca/files/cov/factsheet5-making-ends-meet.PDF. Accessed June 1, 2018.

[CR7] Cloke P (2017). The geographies of food banks in the meantime. Progress in Human Geography.

[CR8] Depa J (2018). Prevalence of food insecurity among food bank users in Germany and its association with population characteristics. Preventive Medicine Reports.

[CR9] Emery JCH (2013). Legislated changes to federal pension income in Canada will adversely affect low income seniors’ health. Preventive Medicine.

[CR10] Feeding America. (2014). *Hunger in America 2014*. http://help.feedingamerica.org/HungerInAmerica/hunger-in-america-2014-full-report.pdf?s_src=W187ORGSC&s_referrer=google&s_subsrc=http%3A%2F%2Fwww.feedingamerica.org%2Fhunger-in-america%2F&_ga=2.59468072.486772252.1531758241-1486936466.1530644975. Accessed June 20, 2017.

[CR11] Fisher A (2017). Big Hunger: The unholy alliance between corporate America and anti-hunger Groups.

[CR12] Food Banks Canada. (2016). *HungerCount 2016*. https://www.foodbankscanada.ca/getmedia/6173994f-8a25-40d9-acdf-660a28e40f37/HungerCount_2016_final_singlepage.pdf. Accessed June 20, 2018.

[CR13] Garratt E (2017). Please sir, I want some more: An exploration of repeat foodbank use. BMC Public Health.

[CR14] Garthwaite K (2015). Food for thought: An ethnographic study of negotiating ill health and food insecurity in a UK foodbank. Social Science and Medicine.

[CR15] Government of Canada. (2016a). *Highlights of the National Shelter Study 2005–2014*. https://www.canada.ca/en/employment-social-development/programs/communities/homelessness/reports-shelter-2014.html#h2.3-h3.5. Accessed June 20, 2017.

[CR16] Government of Canada. (2016b). *Social assistance statistical report: 2009–13*. https://www.canada.ca/en/employment-social-development/services/social-assistance/reports/statistical-2009-2013.html. Accessed July 11, 2018.

[CR17] Government of Canada. (2017a). *EI regular benefits—Overview*. https://www.canada.ca/en/services/benefits/ei/ei-regular-benefit.html. Accessed June 20, 2017.

[CR18] Government of Canada. (2017b). *Employers’ guide to the Government Employees Compensation Act*. https://www.canada.ca/en/employment-social-development/services/health-safety/compensation/geca.html. Accessed June 20, 2017.

[CR19] Government of Canada. (2017c). *Maternity and parental leave benefits*. https://www.canada.ca/en/financial-consumer-agency/services/starting-family/maternity-parental-leave-benefits.html. Accessed Nov 22, 2017.

[CR20] Greater Vancouver Food Bank. (2017). *About us*. https://www.foodbank.bc.ca/about-us/. Accessed November 22, 2017.

[CR21] Holmes, E. (2017). *Food insecurity in Greater Vancouver: A mixed methods exploratory study with food bank members*. University of British Columbia.

[CR22] Holmes E (2018). “Nothing is going to change three months from now”: A mixed methods characterization of food bank use in Greater Vancouver. Social Science & Medicine.

[CR23] Holmes, E., et al. (2018b). Examining food insecurity among food bank members in Greater Vancouver. *Journal of Hunger & Environmental Nutrition*. 10.1080/19320248.2018.1465001.

[CR24] Klein, S., et al. (2008). Living on welfare in BC: Experiences of longer-term “Expected to Work” recipients. Canadian Centre for Policy Alternatives, BC Office.

[CR26] Kuhn R, Culhane DP (1998). Applying cluster analysis to test a typology of homelessness by pattern of shelter utilization: Results from the analysis of administrative data. American Journal of Community Psychology.

[CR27] Lambie-Mumford H (2012). ‘Every town should have one’: Emergency food banking in the UK. Journal of Social Policy.

[CR28] Lightman ES (2008). Globalization, precarious work, and the food bank. Journal of Sociology & Social Welfare.

[CR29] Loopstra R (2018). Rising food bank use in the UK: Sign of a new public health emergency?. Nutrition Bulletin.

[CR30] Loopstra, R., & Lalor, D. (2017). *Financial insecurity, food insecurity, and disability: The profile of people receiving emergency food assistance from The Trussell Trust Foodbank Network in Britain*. Trussell Trust, University of Oxford, King’s College London.

[CR31] Loopstra R (2015). Austerity, sanctions, and the rise of food banks in the UK. BMJ (Clinical research ed).

[CR32] Martin K (2012). Changing the conversation about hunger: The process of developing freshplace. Progress in Community Health Partnerships-Research Education and Action.

[CR33] McBride S, McNutt K (2007). Devolution and neoliberalism in the Canadian welfare state: Ideology, national and international conditioning frameworks, and policy change in British Columbia. Global Social Policy.

[CR34] McCullum C (2005). Evidence-based strategies to build community food security. Journal of the American Dietetic Association.

[CR35] McIntyre L (2016). Reduction of food insecurity among low-income Canadian seniors as a likely impact of a guaranteed annual income. Canadian Public Policy.

[CR36] Middleton G (2018). The experiences and perceptions of food banks amongst users in high-income countries: An international scoping review. Appetite.

[CR37] Poppendieck J (1998). Sweet charity?: Emergency food and the end of entitlement.

[CR38] Prayogo, E., et al. (2017). Who uses foodbanks and why? Exploring the impact of financial strain and adverse life events on food insecurity. *Journal of Public Health*, 1–8. 10.1093/pubmed/fdx133 PMC6186420 29145590

[CR39] Pulkingham J, Béland D, Daigneault PM (2015). Social assistance in British Columbia. Welfare reform in Canada: Provincial social assistance in comparative perspective.

[CR40] Riches G (2002). Food banks and food security: welfare reform, human rights and social policy. Lessons from Canada?. Social Policy & Administration.

[CR41] Riches G (2018). Food bank nations. Poverty, corporate charity and the right to food.

[CR42] Riches G, Silvasti T (2014). First world hunger revisited: Food charity or the right to food?.

[CR43] Riches, G., & Tarasuk, V. (2014). Canada: Thirty years of food charity and public policy neglect. First World Hunger Revisited. In *First world hunger revisited: Food charity or the right to food?* (pp. 42–56).

[CR44] Roncarolo F (2016). Short-term effects of traditional and alternative community interventions to address food insecurity. PLoS ONE.

[CR45] SPARC BC. (2018). *B.C. seniors’ poverty report card*. https://www.uwlm.ca/wp-content/uploads/2018/06/B.C.-Seniors-Poverty-Report-Card.pdf. Accessed July 4, 2018.

[CR46] Tarasuk, V., et al. (2016). *Household food insecurity in Canada, 2014*. Toronto: Research to identify policy options to reduce food insecurity (PROOF). http://proof.utoronto.ca/resources/proof-annual-reports/annual-report-2014/. Accessed June 12, 2017.

[CR47] Tiehen, L. (2002). *Private provision of food aid: The emergency food assistance system*. US Department of Agriculture, Economic Research Service.

[CR48] Vu, T., et al. (2008). *Living on Welfare in BC*. https://www.policyalternatives.ca/sites/default/files/uploads/publications/BC_Office_Pubs/bc_2008/bc_LoW_full_web.pdf. Accessed July 9, 2018.

[CR49] Wakefield S (2012). Sweet charity, revisited: Organizational responses to food insecurity in Hamilton and Toronto, Canada. Critical Social Policy.

[CR50] Wallace, B., & Klein, S. (2006). *Denied assistance, closing the front door on welfare in BC Vancouver, Canada*. CCPA (Canadian Centre for Policy Alternatives) & VIPIRG (Vancouver Island Public Interest Research Group). https://www.policyalternatives.ca/sites/default/files/uploads/publications/BC_Office_Pubs/bc_2006/denied_assistance.pdf. Accessed July 9, 2018.

